# Patterns of physical activity in hunter-gatherer children compared with US and UK children

**DOI:** 10.1038/s41598-024-81326-w

**Published:** 2025-01-30

**Authors:** Luke Kretschmer, Mark Dyble, Nikhil Chaudhary, David Bann, Gul Deniz Salali

**Affiliations:** 1 Centre for Longitudinal Studies, Social Research Institute, UCL, London, UK; 2 Department of Anthropology, University College London, 14 Taviton Street, London, UK; 3 Department of Archaeology, Leverhulme Centre for Human Evolutionary Studies, University of Cambridge, Cambridge, England

**Keywords:** Anthropology, Public health

## Abstract

Contemporary hunter-gatherers are highly active, but little is known about physical activity levels in hunter-gatherer children. We analysed 150 days of accelerometer data from 51 BaYaka hunter-gatherer children (aged 3–18) in the Republic of Congo, comparing it with British and American children using samples from Millennium Cohort Study (MCS) and National Health and Nutrition Examination Survey (NHANES). BaYaka children were highly active, engaging in over 3 h of moderate-to-vigorous physical activity (MVPA) daily, surpassing British adolescents by over 70 min. Unlike US children, whose activity diminished with age, BaYaka children’s activity levels increased, irrespective of gender. This trend suggests that formal education may suppress activity among American children, a pattern not seen in the BaYaka community. Reflecting their foraging lifestyle, activity patterns varied within and between days in BaYaka children, a contrast to the more uniform daily activity observed in American children. Furthermore, our data challenges the concept of ‘teenage chronotypes’ prevalent in post-industrial societies, with adolescent BaYaka maintaining shorter sleep phases and later bedtimes, synchronized with sunrise. These findings highlight the impact of a foraging upbringing on children’s activity levels, providing a benchmark for understanding childhood physical activity and wellbeing.

## Introduction

With physical activity associated with increased lifespan and health span^1,2,3^there is significant interest in examining the cultural factors driving increased activity. However, research that has attempted to do this has largely done so using culturally similar, highly sedentary, high-income populations^4^. These populations represent a narrow fraction of human cultures, for whom daily calorie acquisition is decreasingly dependent on physical activity, with labour increasingly sedentary, and foraged foods replaced with market-bought goods^5^. Research on children in high-income populations showed that despite the well-documented benefits of physical activity for healthy childhood development^3,6^, up to 80% of children globally fail to meet recommended physical activity guidelines^7^. Given the low rates of physical activity among children in these populations and the importance of movement for lifelong health^8,9,10^, it is important to establish comparative models of childhood physical activity. Cross-cultural research on subsistence populations, including agriculturalists, horticulturalists, pastoralists, and hunter-gatherers, offer a valuable perspective as they reflect the diversity and history of humankind. Of these, hunter-gatherers may provide the best approximation of the physical activity patterns of the foraging lifestyle that dominated most of human evolutionary history^11^.

Notably, hunter-gatherer populations appear to have exceptionally low rates of non-communicable ‘diseases of modern life’, including low rates of obesity, type II diabetes, hypertension, and auto-immune disorders^4,11,12,13^. As such, any transferable insights may help inform meaningful interventions in post-industrial populations. Studies of adults in subsistence populations reveal volumes of activity in excess of those seen in high-income populations^14,15,16,17^. For example, a study by Raichlen et al., (2017)^18^of Hadza hunter-gatherers of Tanzania observed daily amounts of moderate to vigorous physical activity (MVPA) to be in excess of weekly observations for US adults (945 min/week vs. 64 min/week) with similarly high volumes seen in other subsistence populations^16,19,20^. Given that the volumes of activity an individual engages in as they exit adolescence often predicts their volume of activity throughout adulthood^21^, we need further cross-cultural studies on children’s physical activity levels. However, a detailed understanding of the activity patterns of hunter-gatherer children is lacking.

According to embodied capital theory, human childhood has evolved to allow necessary time to develop complex skills needed for the hunting and gathering niche^22^. As such, hunter-gatherer childhoods are marked by the learning of skills such as gathering wild plants, hunting animals, collecting honey and caterpillars, fishing, childcare and domestic activities^23,24,25^. These skills often involve physically demanding activities such as climbing trees, using knives and machetes for digging and cutting, walking long distances in difficult terrain and carrying heavy loads including water and firewood. Although there is gendered division of labour, both boys and girls engage in laborious activities^23,26^. Hence, it is possible that, unlike high-income populations where boys often undertake greater volumes of activity^27^, hunter-gatherer boys and girls may engage in similar levels of activity. Forager children start practising these skills from infancy, first by imitating and observing others, then by play and practice in mixed-aged autonomous children’s groups, often in the absence of adults^23,28^. Moreover, children also regularly act as caregivers, with children as young as 4 years old helping to care for babies, regularly holding and carrying them^29,30,31^. Their foraging skills increase with age and with respect to the resource exploited^32^. Therefore, the age-related patterns of physical activity may divert from those observed in high-income populations. Research frequently observes that children in high-income populations engage in diminishing volumes of physical activity with age^33^, with notable reductions in activity following the start of formal schooling^34,35,36,37^and following puberty^37,38^, with boys commonly undertaking more physical activity than girls^39,40^.

From the limited research detailing hunter-gatherer children’s activity levels, a study of space use in Baka children of Cameroon using waist-worn accelerometers observed that children undertake over 20,000 steps a day, with daily distances increasing with age^41^. Another, based on observational data, estimated an increase in moderate and vigorous intensity activity with age in Hadza children^42^. In contrast, amongst the Tsimane forager-farmers, physical activity was observed to decrease with age amongst adolescents^43^. There are, however, gaps in the current literature on hunter-gatherer children’s activity levels: (1) we lack direct comparisons of activity across cultures, (2) it is unclear whether boys and girls differ in their levels of activity as often observed in high-income populations, (3) the daily and hourly variation in activity levels are not well documented. In the absence of a regular routine of sedentary and active behaviours provided by classrooms, hunter-gatherer children may have more opportunity to vary in their activity within and between days, (4) no research among hunter-gatherers has directly examined the role of day length, though changes in daily activity may be explained by variations in the length of the wakeful day. Similar trends have been observed in post-industrial populations, where children spend less time asleep as they age^44^.

To address the paucity of data on physical activity among hunter-gatherer children, we examined patterns of physical activity among BaYaka children from the Republic of Congo using wrist-worn triaxial accelerometers. As well as examining patterns of activity, we additionally assessed differences in the sleep window (time of onset and wake-up) to examine differences in the length of waking days. We compared patterns of activity between BaYaka children, with two nationally representative studies from high-income countries, the American National Health And Nutrition Examination Survey (NHANES) and the British Millennium Cohort Study (MCS). We predict higher levels of physical activity, reduced gender differences in activity levels, and greater variability in daily activity levels in hunter-gatherer children compared to the children in the UK and the US.

## Methods

### Study population

Data for the present study was collected amongst the Mbendjele BaYaka of the Republic of Congo. The BaYaka are a group of hunter-gatherers residing in the rainforests of the Congo Basin. The Mbendjele are a subgroup of this population who speak Mbendjele^45^. The BaYaka live in multifamily camps, consisting of 10–60 individuals^46^, and move campsites based on the availability of forest products and trading opportunities throughout the year. The mode of subsistence is largely dependent on forest products: with food obtained by hunting, fishing and gathering wild products such as yams, caterpillars and honey depending on the season. This can be supplemented by trade with farmers and passing traders, with whom forest products are exchanged for market goods and cultivated foods^47^. The children in the study population do not attend school or receive formal education.

### Fieldwork and data collection

The BaYaka’s physical activity data were collected during fieldtrips in 2018 and 2022 in the Likouala region of Congo’s Ndoki swamp forest. The 2018 fieldwork took place in July and August, when the main subsistence activities involved caterpillar and honey collecting, hunting, collecting wild yams and plants and occasional fishing. The 2022 fieldwork took place during fishing season in February when BaYaka move to deeper parts of the forest (leaving their camps set up alongside mud roads) and establish camps close to river streams to engage in fishing.

Between the two fieldtrips, 71 children aged up to 19 years wore a GENEActiv accelerometer device on their non-dominant arm. In 2018 46 children (24 boys) wore the device for up to two consecutive days. In 2022, 24 children (12 Boys) wore the device for 6 consecutive days. Children were excluded from the present analysis if they were aged under 3 years (n = 6), if their accelerometry data were corrupted during recording (n = 1) or if they did not have at least one complete day (midnight-midnight) of recording (n = 13). Together, this left a sample of 51 children (2018 = 28, Total Boys = 29) totalling 150 days of activity recording.

Prior to distribution devices were set to run at 100 Hz, with participants instructed on how to wear the device, and that they should wear the device continuously throughout the recording. Devices were waterproof and worn during all activities. Age estimates for participants were established using a Bayesian method using relative age ranks based on those with known ages^48^.

### Physical activity metrics

Accelerometer recordings were processed using the R package GGIR^49,50^. The maximum recording length was set to 7 days from the start of recording. While no restriction on excluding the first or last day of study was stipulated, this was included de facto by excluding all incomplete days. Activity was calculated in 24-h windows from midnight to midnight, based on West Africa time (UTC + 1 h).

Acceleration is calculated as milligravitational units, minus one g to account for gravity (mg ENMO). Accelerations are presented as a continuous measure across the recording (mean mg ENMO) and subset into standard thresholds (Sedentary ≤ 50 mg < Light ≤ 100 mg < Moderate ≤ 400 mg < Vigorous) consistent with previous research^51,52^. MVPA is a sum of all the minutes in moderate or vigorous intensities. Sedentary is presented as all wakeful minutes below the threshold of 50 mg ENMO^53^. Thresholds are presented as an unbouted measure, summing all time per day within an intensity threshold. Physical activity data for all individuals was collated for each individual at a daily level (1 entry per person per day of recording) and an hourly level (1 entry per person per hour of the day).

Sleep was detected by GGIR as periods of time in which the device was in a sustained, inactive state^54^. This is classified as any block of 10 min, in which the average movement of the device is less than 5° in any axis over each 5-s epoch. The algorithm defines the longest sustained inactive bout within each day as sleep, allowing for the sleep bout to be broken for up to one hour before separating it into two bouts of sustained inactivity. From this, the estimated onset and end of sleep windows can be calculated from the final and first timepoint in each day in which an individual was not in a device defined bout of sleep. Sedentary activity was calculated by removing all daily time in device detected sleep from the daily accumulated time below 50 mg ENMO.

No periods of non-wear were detected during the study period. The only device detected non-wear (defined by the device moving less than 5 degrees in at least 2 planes in a rolling 10-min window)^49^ occurred on an individual’s final day of recording, representing the time between removing the device and stopping the recording and was excluded from the analysis.

### Comparison populations

We used two publicly available datasets from high-income populations that had also employed wrist-worn accelerometers. One was the National Health and Nutrition Examination Survey (NHANES) and the other was the 6^th^ sweep of the UK Millennium Cohort Study (MCS). We selected these two datasets for their complementary strengths: NHANES enables comparisons of activity levels across different age groups, while MCS allows for direct comparison of absolute values. Below, we provide a detailed explanation of the properties of these datasets and our rationale for choosing them as the basis for our comparisons.

NHANES is a repeated cross-sectional study conducted across the United States and designed to be broadly representative of the US population, with some oversampling of minority groups to ensure statistical power if subset^55^. The study was conducted in 2013 to 2014 with individuals aged 3 years or older wearing a wrist-worn triaxial accelerometer (Actigraph GT3X +) for 7 continuous days on their non-dominant arm. We used a subset of this data to match the age range of the BaYaka sample. Prior to making the data available the NHANES data holders flagged periods of device non-wear so that they could be removed from the dataset^56^. Only complete days were included in the analysis, resulting in a mean of 5 days complete days of recording per person. The included NHANES sample contained 2391 children (1200 boys) aged between 5 and 18 years The mean age was 11 years 8 months and was similar between boys and girls (boys: 10.60 years; girls: 10.76 years). Of the individuals included, 25% identified as non-Hispanic White, 25% and non-Hispanic Black, 25% as Mexican American, 10% as other Hispanic and 15% as ‘other’ ethnicity. The mean MIMS-Unit (Monitor Independent Motion Sensing) was 14,673 and was similar between boys and girls (boys: 14,534 MIMS; girls: 14,813 MIMS).

We used NHANES data solely to compare activity trajectories across ages, not absolute values. NHANES employed a different output metric (high-pass filtered) on a comparable wrist-worn device (Actigraph GT3X-plus)^57,58^. Both the device used in this study and the device used at NHANES capture the same body movements but the output metrics are different. Due to this difference, we did not compare the volumes of activity directly between NHANES and the BaYaka. Instead, internally estimated z-scored average activity measures were used to examine variation across ages^56^.

The MCS accelerometry data included 4,533 individuals (2,182 boys) aged 14 years (Table S3)^59,60^. Since the data were collected using the same device as the BaYaka dataset, we were able to make direct comparisons among the 14-year-olds. Data processing was conducted using GGIR, consistent with the approach taken for the BaYaka data, with average accelerations and time spent in MVPA calculated and presented in the same units. Non-wear periods in the MCS were identified and managed in the same way as in the BaYaka dataset^60,61^.

To ensure comparability, the cut points applied in this study were aligned with those used in the MCS, which included all time within thresholds without being confined to bouts. As of this writing, no universally agreed set of cut points exists for multi-age cohorts using GENEActiv devices, nor has one been validated for populations such as the BaYaka. Roscoe et al. (2017)^62^ identified similar cut points among preschoolers as those used for the 14-year-olds in the Millennium Cohort Study, suggesting that the impact of age on time spent within thresholds may be minimal. To mitigate potential limitations associated with cut points, we primarily focused on mean accelerations for comparisons, using thresholds mainly for illustrative purposes.

MCS data allowed for absolute value comparisons, as the methods and devices employed in the MCS matched those used in our study. However, the MCS has several limitations: (1) all participants are the same age-14 years old, (2) hour-by-hour measures are unavailable, and (3) activity was recorded for only two days. Due to these limitations, we used NHANES for age and daily variation comparisons.

### Statistical analyses

#### Modelling BaYaka activity

To model difference between ages and genders, linear mixed effect models were employed, using the lme4 package in R^50,63^. Outcome variables were either the mean acceleration across the day or the volume of time in a given threshold of activity. Models were adjusted for age and gender, both as additive effects, and with an interaction term to allow gender specific slopes for age. Additionally, year of recording was included in the models to account for potential seasonal differences between the two visits to the field, with the 2022 data collected during fishing season, while the 2018 data were collected during caterpillar season. Individual ID was included as a random effect to adjust for clustering of values within an individual across the days of recording. The sample was restricted to include only days with a complete 24 h of recording, and to individuals who had complete data for the included covariates. This left a sample of 51 individuals and a total of 150 person-days of activity data.

Mixed effect models were also used to analyse differences in activity across the day. Mean accelerations were averaged over 3-h windows, for each day of recording for each child. The effect of time of day (in 3-h windows) was modelled against the mean acceleration in that window, with the fixed covariates of age and gender included. To account for the nested clustering of results within days and within individuals, random effects of day and individual ID were included to reflect this nesting of groups. Because 2022 data included a longer period, we used this data for analysis of within-day variation in activity. The reference in these models mirrors previous models for overall activity, with the reference time window being in the middle of the sleep window (midnight – 3am). Three-hour windows were used to improve the contrast between windows of time and reduce the number of pairwise comparisons.

To model differences in wake-up times and sleep-onset, data from the 2022 data collection was used. With recording conducted over the same week for all individuals, light conditions were the same for all individuals. For each of wake-up and sleep onset, the outcome of time of day is modelled against the effect of age and gender as fixed effects with the day of recording nested within each individual as a random effect.

#### Modelling patterns of activity in the BaYaka and the US sample

To compare the BaYaka and NHANES study, the mean acceleration on each day of recording (measured as mg ENMO & MIMS Unit) was standardised using internally calculated z-scores. Z-scored mean accelerations were then employed in mixed effect linear models adjusted for age, gender and population as fixed effects, with the individual ID included as a random effect. To examine differences between populations in the slope with age, an interaction term was included between age and population.

## Results

### BaYaka children undertake three times more activity than WHO recommendations.

**Fig. 1:**
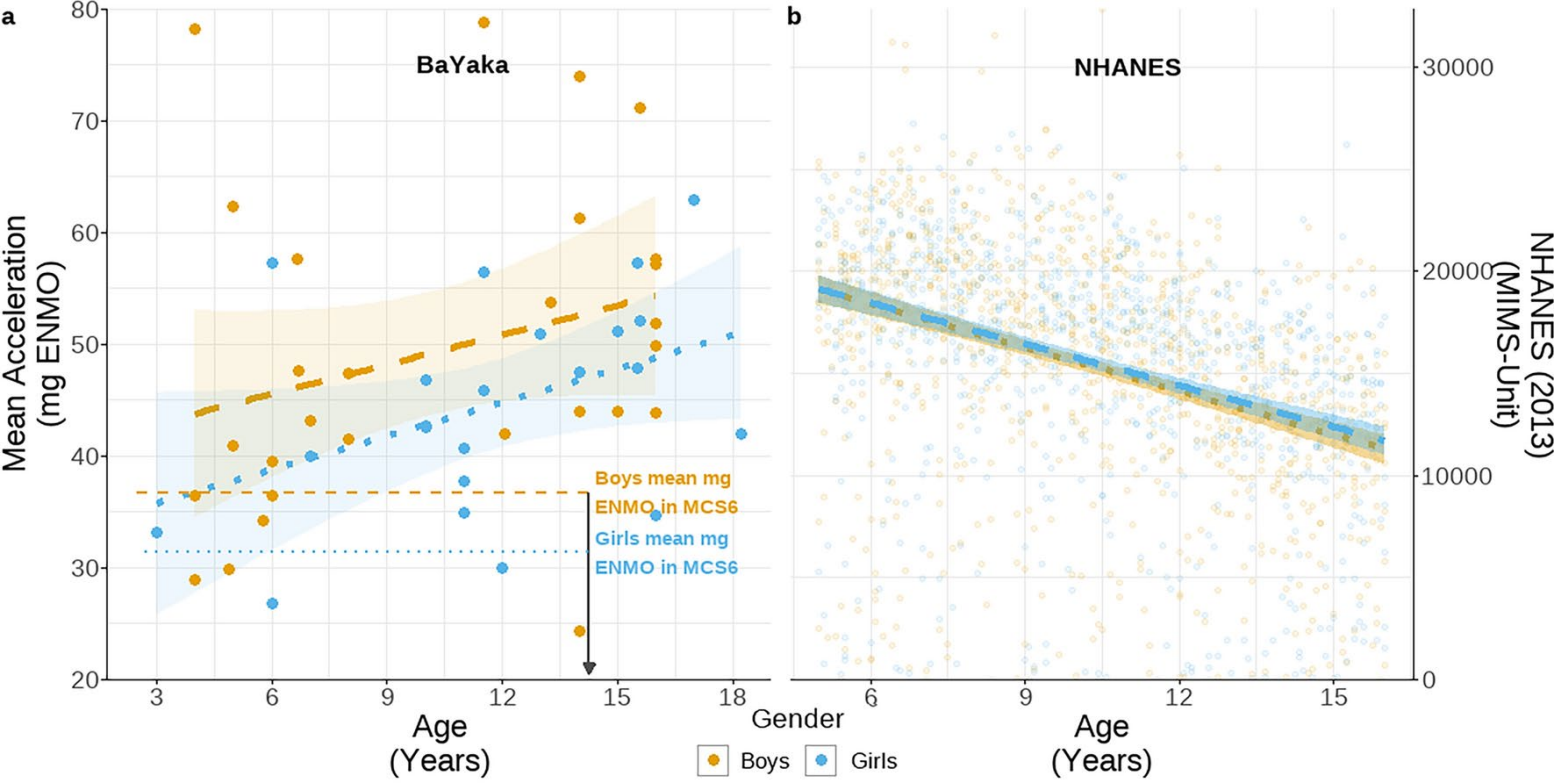
Mean acceleration amongst (**a**) BaYaka children as measued by mg ENMO and (**b**) US children in the 2013 NHANES study measured in MIMS-Unit. For both samples, all individuals aged between 3 and 18 years are included. Orange dots represent boys, blue dots represent girls. The fitted trend lines represent the linear model estimates and 95% CI as outlined in. Horizontal dashed (boys) and dotted (girls) in panel a represent mean volumes observed in the Millennium Cohort Study (MCS6) where individuals were 14 years old. The Millennium Cohort Study processed physical activity in a manner consistent to that employed here, facilitating direct comparison among the 14-year-olds. Note that it is not possible to make direct comparisons of volumes of activity with the NHANES data due to the difference in the measurement unit.

**Fig. 2:**
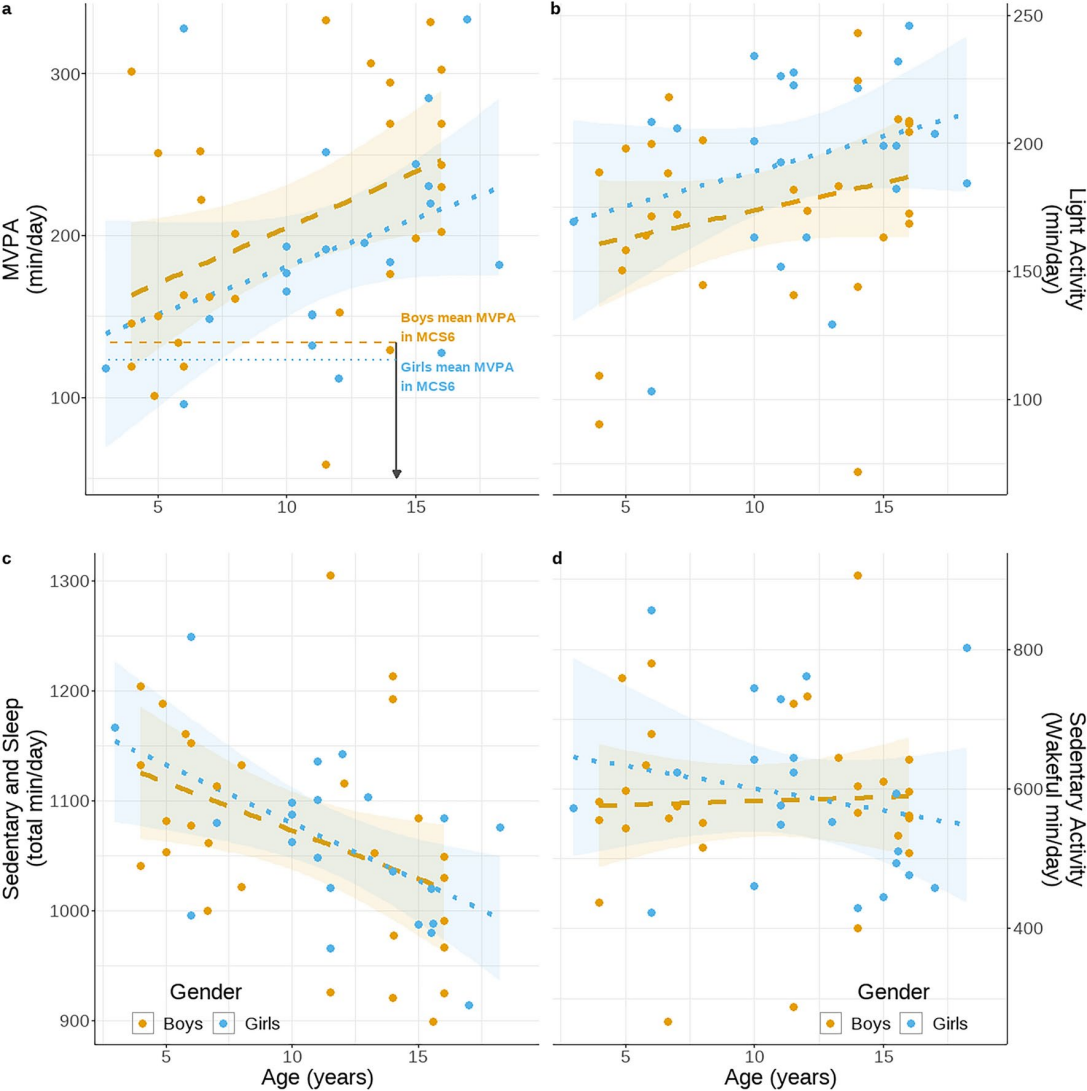
Mean volumes of activity per day by age. Boys are shown in orange and girls in blue (**a**) volume of moderate-to-vigorous intensity physical activity (mg ENMO > 100 mg) (**b**) volume of light intensity activity (mg ENMO: 50–99 mg), (**c**) volume of low intensity activity including sleep (mg ENMO < 50 mg), (**d**) volume of wakeful sedentary activity (mg ENMO < 50 mg). In panel a, the horizontal dashed (boys) and dotted (girls) represent mean volumes observed in the Millennium Cohort Study (MCS6) where individuals were 14 years old. The Millennium Cohort Study processed physical activity in a manner consistent to that employed here, facilitating direct comparison. Only values for MVPA and mg ENMO have been made available from MCS6. The volume of MVPA here includes all minutes above the threshold of 100 mg ENMO. The fitted trend lines represent the linear model estimates and 95% CI as outlined in.

Compared to the sampled high-income populations, BaYaka children were exceptionally active. Across the sample of 51 children (29 boys), the average day included over three hours of MVPA (Boys: 206 min/day, Girls: 192 min/day), a volume that is triple the WHO recommendations for children^3^. Approximately twenty minutes of this time were spent in vigorous activity (see Table S1). The average 24-h period included a further three hours of light intensity activity, over 9 h spent sedentary with approximately 8 h spent asleep. Together, this resulted in a mean acceleration of 47 mg ENMO (Boys: 48 mg; Girls: 45 mg;). Compared to the average physical activity level of the British 14-year-olds in the UK MCS dataset, amongst the five 14-year-old BaYaka the mean acceleration was 16 mg higher, with volumes of MVPA 65% greater (Figs. 1 & 2).

### BaYaka children, regardless of gender, get more active with age unlike the children in the UK and the US

Overall activity increased with age in the BaYaka (+ 0.9 mg/year, CI: 0.1 – 1.6), with no observed difference between boys and girls (Girl: −4.5 mg, CI: −11.1 – 2.1;). In both boys and girls activity was approximately 1 mg higher for each additional year of age, maintaining a consistent trajectory between them (Fig. 1). In contrast, the children in the US NHANES sample were more active in early childhood, and their activity declined with increasing age (Fig. 1). Owing to differing means of reporting accelerations, internally calculated z-scored daily accelerations are used to assess differences between American and BaYaka children. Mean accelerations in the NHANES sample decreased with age by 0.51 standard deviations for each standard deviation of age (CI: −0.75—−0.27), while BaYaka accelerations increased by 0.22 standard deviations for each standard deviation of age (CI: −0.01 – 0.46).

Separating overall activity in the BaYaka into the standard activity thresholds, the increase in mean acceleration was driven by greater volumes of moderate to vigorous intensity activities which in turn were offset by a decrease in sedentary activity and sleep (Fig. 2). Volumes of MVPA were similar for both boys and girls (Girls: −24.1 min/day, CI: −56.1 – 8.0), increasing by 8 min per day for each additional year of age (CI: 4.2 –11.7). Offsetting this, volumes of both sleep and sedentary (< 50 mg) decreased by an estimated 10 min per day for each additional year of age (CI: −14.8−−3.4) (Table S4), with no statistically significant difference between boys and girls (Girls: + 7.6 min/day, CI: −34.8 – 49.9). Of this, volumes of wakeful sedentary behaviour appear to be more stable with age, (−5 min/day CI: −13.4 – 2.7) indicating that differences in total low intensity activity (Sleep and Sedentary, < 50 mg) with age were also driven by changes in the sleep phase; the time between sleep onset and wake-up. As with sedentary behaviours, there were minimal changes to the estimated volumes of light intensity activity with age (+ 2.5 min/day, CI: 0.2 – 4.8).

### Older children stay up later, but everyone wakes up together.

**Fig. 3:**
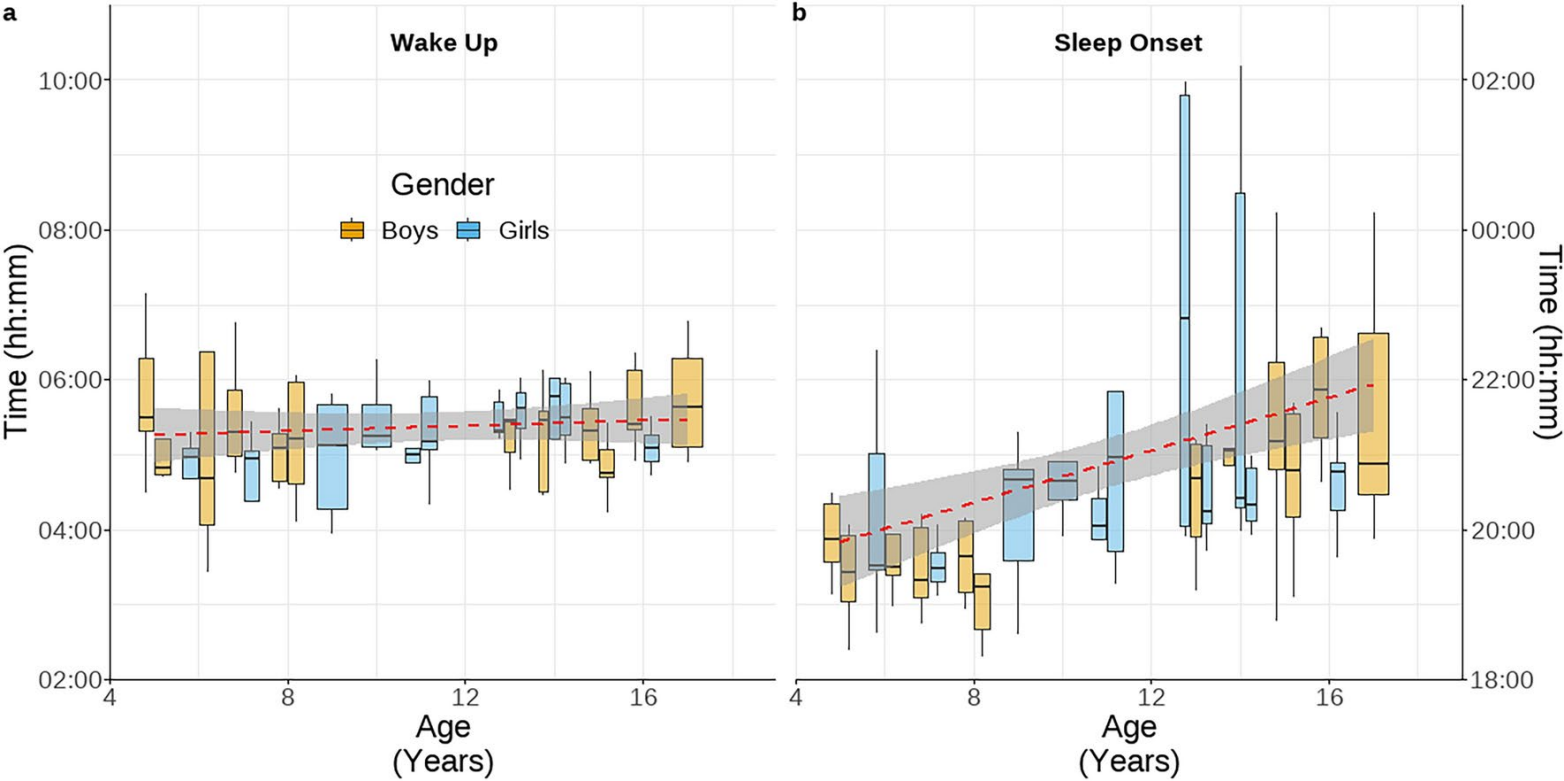
Device estimated (**a**) wake up and (**b**) sleep onset times across individuals. Dashed red line represents the estimate from linear model. Y-axes are matched in length to 9 h each to aid interpretation. Sleep is classified by GGIR as any block of 10 min, in which the average movement of the device is less than 5° in any axis over each 5 s epoch. The algorithm defines the longest sustained inactive bout within each day as sleep, allowing for the sleep bout to be disrupted for up to one hour before. From this the estimated onset and end of sleep windows can be calculated as the final and first timepoint in each day in which an individual was not in a device defined bout of sleep.

Interestingly, BaYaka children had little variation in their wake-up time (+ 4 min/year, CI: −5 – 13), but their time of sleep onset was later with increasing age (+ 10 min/year, CI: 2 – 19) (Fig. 3), with no difference between boys and girls (Table S6). The reduced time in bed may partially underlie the reduction in low intensity activity (Sleep and sedentary, < 50 mg) with age, with sleep onset beginning 10 min later per year of age, which contributes to the decrease of 10 min a day of low intensity activity (Table S6).

### Diurnal activity patterns may reflect children’s foraging activities.

**Fig. 4:**
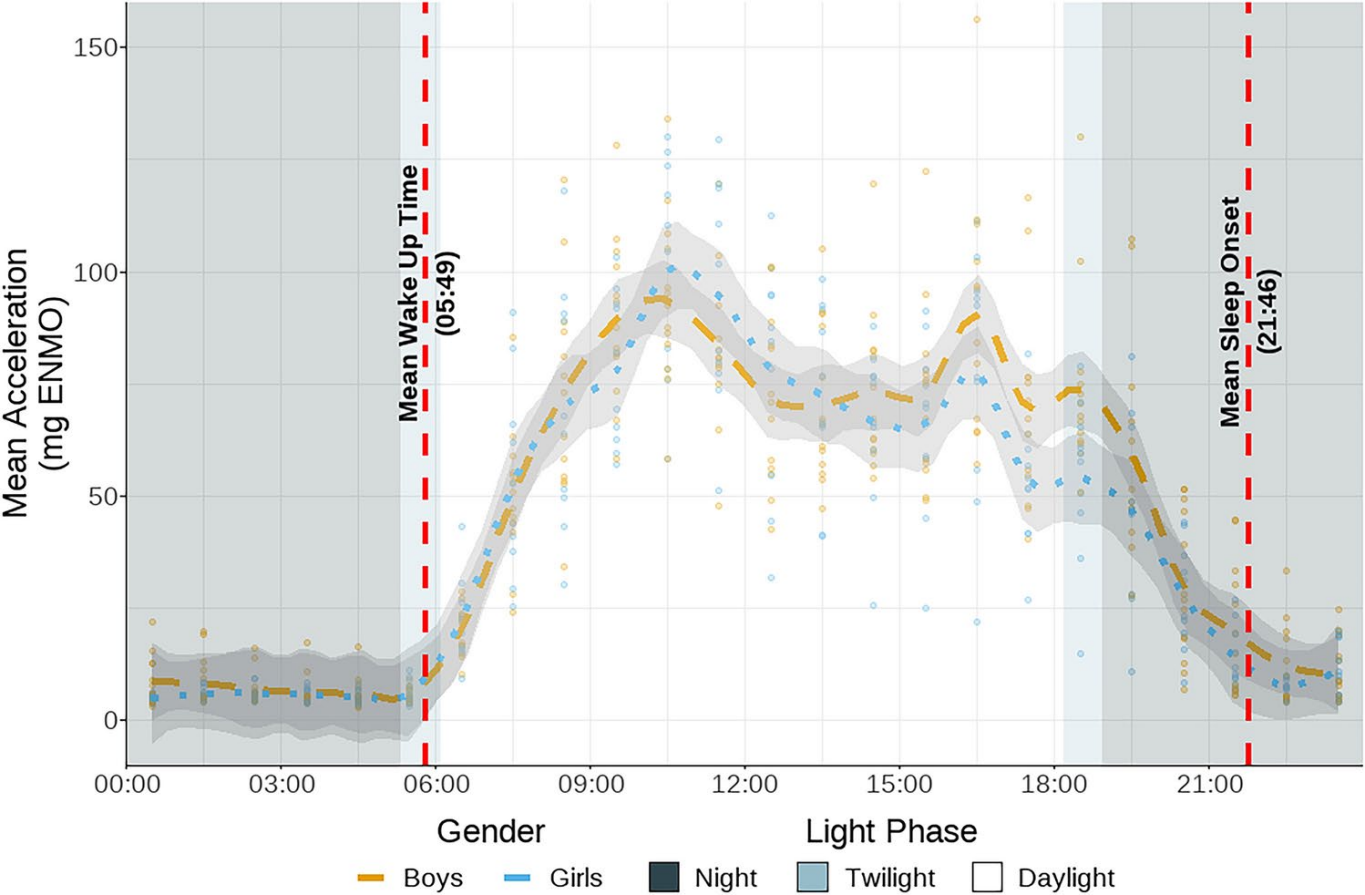
Distribution of physical activity throughout the day. For each individual in the 2022 data, their mean acceleration at each hour averaged across the 5 days of recording is presented as a single point, with colour corresponding to gender. The mean acceleration was calculated for each hour interval and presented at the half hour (i.e., the mean acceleration between 09:00 and 10:00 is presented at 09:30). Presented light phases of night, (dusk to dawn; 18:56–05:20), Twilight (dawn to sunrise; 05:20–06:06 & sunset to dusk; 18:11–18:56), and Daylight (sunrise to sunset; 06:06–18:11) were taken from the nearest town on the first day of recording. The dashed horizontal lines represent the mean time of wake (05:49) and sleep onset (21:46) across the sample presented in 24 h hh:mm format.

BaYaka children’s activity patterns follow a clear diurnal pattern during the fishing season (Fig. 4). Children were at their least active overnight, between 21:00 and 06:00, during which the average intensity of acceleration was below 10 mg Transitioning from sleep, individuals rose to modest levels of activity in the early morning (06:00 to 09:00: 40 mg) before being at their most active in the late morning (09:00 to 12:00: 80 mg; Table S7). During this most active period, children were twice as active as the previous time window. Throughout the afternoon (12:00 to 18:00) activity remained high and was stable at around 65 mg. Following sunset, activity levels declined into the sleep window, with a mean acceleration of 42 mg between 18:00 and 21:00. The two peaks in activity, one during early morning and the other late afternoon, likely reflect the walks children undertook when going into the forest for fishing and returning to the campsite after foraging (Fig. 4). While children in the NHANES sample also undertook most of their activity during the day, compared to BaYaka, US children appeared to vary more in the times at which they woke-up, and remained active further into the night.

### Activity levels in BaYaka children were more varied between days compared to the US children.

Compared to US children in the NHANES dataset, BaYaka children exhibited more variation in how much activity they undertook each day. Using intercept-only mixed effect models on standardised mean accelerations amongst BaYaka children with five days of recording (N = 23) to match the length of the recording in NHANES, a lower intra-class correlation (ICC) (0.50) was observed for BaYaka children than US children (0.66) (Table S9, Figure S2). ICC measures the variability within a group (here an individual’s ID) between 0 and 1, with scores closer to 0 indicating greater within-individual variation.

## Discussion

The BaYaka children included here were highly active, engaging in over three hours of MVPA per day on average, a figure three times higher than the WHO recommendations for children^6^. Amongst the BaYaka, older children were more active than younger children, with greater mean accelerations driven by more time in MVPA (+ 8 min/day for each year of age) exchanged with some sedentary activity and sleep time (−10 min/day for each year of age). This is in stark contrast to children in the US sample presented here and other high-income populations where volumes of physical activity commonly peak in early childhood (ages 5 to 6) and decline from then, until reaching a low plateau in adulthood^21^. Observational data amongst Hadza children also suggests an increase in MVPA with age^42^, and using a device measure here we observed BaYaka adolescents to engage in over 90 min more MVPA per day than British adolescents. Unlike those in the British cohort, no difference in activity was observed between BaYaka boys and girls, and this did not change with age. While adolescent BaYaka went to bed later, all children appeared to wake up together in time with sunrise. Moreover, BaYaka children showed more daily variation in their activity levels compared to the children in the US with their hourly activity patterns reflecting their foraging lifestyle. These results highlight the role of subsistence mode and potentially highlight the negative effect of formal schooling on children’s activity levels.

High levels of activity were observed from a young age, reinforcing observations that BaYaka children start practicing physically demanding foraging skills from early childhood^23,25^. Early childhood in hunter-gatherer and mixed foraging populations is marked by significant playtime in which children practice foraging, improve skills, and contribute to the family economy, either directly through food acquisition or indirectly through domestic work that frees other individuals to forage^23,30,64^. Forager playgroups are critical for skill acquisition, often mimicking adult activities. For example, during 2022 fieldwork, we observed children aged 3–12 practicing dam fishing independently. This play and practice typically peak at age five for BaYaka children, then gradually shift towards work as they approach adolescence^23,64^. Activity increasing across childhood and adolescence mirrors the embodied capital theory in which a child’s skill, efficiency and the range of goods targeted increases as they grow^22,65^. Conversely, post-industrial populations see decreased activity over the same age span^37^. A core difference between these children is in the behaviours they are increasingly modelling with age; for hunter-gatherer’s the archetypal adult is highly active, while parents in post-industrial populations are often less active than their children^26,56^. With the association between adolescent and adult activity, efforts to address adolescent activity may have lasting effects throughout adulthood.

BaYaka hunter-gatherers exhibit gendered division of labour with women targeting gatherable goods while men prioritise hunting and collecting honey. This division is reflected in children’s behaviour through their activities and play^64^. Despite the gendered division of labour and differences in the timing of the transition from play to work, our findings indicate that mean physical activity did not differ significantly between BaYaka boys and girls at all ages. This contrasts with the MCS study included here and other studies in industrialised populations, where boys often engage in higher levels of physical activity^34,36,39,40,66,67,68,69,70,71^. This suggests that observed differences in activity between boys and girls are of cultural rather than biological origin^72^.

Although we did not find significant differences, our data suggest a trend where mean acceleration may be higher in boys than girls throughout childhood. With larger sample sizes, these trends could potentially reach statistical significance. Sample size is a common limitation in many hunter-gatherer studies, as most research on mobile, small-scale societies like the BaYaka inherently involves small samples. Consequently, most device-based physical activity studies among foragers report sample sizes of fewer than 50 individuals across all ages^12,19,73^, which limits the statistical power to detect subtle differences between groups.

Our data collection has certain limitations. The 2018 data collection was limited to two days of recording. To address this, we excluded the 2018 data from analyses examining within-day and between-day patterns. However, for other analyses, we included the 2018 data to maximize sample size, as no meaningful differences in activity were observed between the two-day and six-day recordings (see SI Table S4, where a linear mixed-effects model incorporates the 2018 sample statistics as a control variable). Including this data strengthens the study by contributing to one of the largest sample sizes in any research on hunter-gatherer physical activity, particularly those focusing on children.

Seasonality plays a significant role in the subsistence activities of the BaYaka, as the availability of food sources changes with the seasons^74^. There are two main seasons in Northern Congo: wet and dry. The 2022 fieldwork was done in dry season when BaYaka move to deeper parts of the forest and establish camps close to river streams to engage in fishing^75^. During our fieldwork, BaYaka children frequently practiced dam fishing which involves making dams in the streams, bailing out the water, before killing the fish with machetes. The 2018 fieldwork occurred in July–August, a wetter period during which children engaged in treks into the forest to forage for caterpillars while they were abundant. Despite the seasonal differences in foraging activities, our results suggest that the required activity levels for engaging in those are similar (Table S4). Our hourly analysis also highlighted the diurnal trends in BaYaka children’s foraging, which mimicked adult foraging. The day was active with peaks of activity in late morning and early evening, coinciding with extended periods of walking to and from the forest.

The observations in the BaYaka and the sampled high-income populations suggest that formal schooling may promote inactivity in children, limiting their autonomy by mandating long periods of sedentary activity^37,76,77,78,79^. This schooling structure might contribute to increasing mental health problems and decreased child happiness observed in high-income populations^79,80^. Without formal classrooms, BaYaka children choose their activities, with no imposed sedentary behaviour. Their daily activities, unlike American children’s regimented school schedules, are more variable and reflect an ecology of autonomous play, foraging, and rest. The structured, sedentary periods may impact children’s health and wellbeing, including adiposity increases^81^. Schooling may also have a lasting effect on activity, with observation amongst Hadza adults that men that received more years of formal schooling undertook less MVPA per day^17^. Implementing BaYaka perspectives, like breaking up prolonged bouts of sedentary behaviour as employed in forest-school and Udeskole (outdoor-school) programs has been observed to increase overall activity^82,83^.

A teenage chronotype has been proposed to underlie later sleep onsets and wake up times amongst adolescents^84^. Similar to studies in post-industrial populations, adolescent BaYaka did enter the sleep phase at a later time, possibly reflecting a reduction in sleep requirements with age^44,85^. However, there was little variation in wake-up times. For both, light is likely important; in the evening campfires allow the extension of wakeful activity safely into the night^86^but do so without the blue wavelength light associated with disrupted sleep in industrialised populations^87^. Then in the morning, with little ability to completely exclude light, daylight appears to be the entraining factor for waking up, as has previously been observed in Hadza adults^88^.

## Conclusion

Highlighting the contemporary suppression of childhood physical activity in high-income populations, BaYaka children were highly active, engaging in over three times the WHO recommendations for physical activity. In further contrast to high-income populations, physical activity was more variable across days and increased with age for boys and girls. Utilising cross-cultural studies to examine the impact of low childhood activity on health, wellbeing, and development, with highly active hunter-gatherer populations as a reference point, may offer richer insights than comparisons with less active groups.

## Supplementary Information

Supplementary Information.

## Data Availability

Data is available through the Open Science Framework repository: https://osf.io/9q523/?view_only = c15d8c61cf7b4efab487be149ea409ae 79. A copy of the R code used is uploaded within the OSF repository. Details and access to the NHANES dataset can be found here (https://www.cdc.gov/nchs/nhanes/index.htm). Detail on the Millennium Cohort Study can be found here (https://cls.ucl.ac.uk/cls-studies/millennium-cohort-study/ ) and can be accessed via the UK data service.
